# Honokiol ameliorates cigarette smoke‐induced damage of airway epithelial cells via the SIRT3/SOD2 signalling pathway

**DOI:** 10.1111/jcmm.17981

**Published:** 2023-10-05

**Authors:** Fei Li, Chunyu Ye, Xiuli Wang, Xinting Li, Xiaoxia Wang

**Affiliations:** ^1^ Department of Pulmonary and Critical Care Medicine Shanxi Provincial People's Hospital Taiyuan China; ^2^ The Fifth Clinical Medical College of Shanxi Medical University Taiyuan China; ^3^ Department of Biochemistry and Molecular Biology Shanxi Medical University Taiyuan China

**Keywords:** cigarette smoke, COPD, honokiol, inflammation, SIRT3

## Abstract

Cigarette smoking can cause damage of airway epithelial cells and contribute to chronic obstructive pulmonary disease (COPD). Honokiol is originally isolated from Magnolia obovata with multiple biological activities. Here, we investigated the protective effects of honokiol on cigarette smoke extract (CSE)‐induced injury of BEAS‐2B cells. BEAS‐2B cells were treated with 300 mg/L CSE to construct an in vitro cell injury model, and cells were further treated with 2, 5 and 10 μM honokiol, then cell viability and LDH leakage were analysed by CCK‐8 and LDH assay kits, respectively. Apoptosis was detected by flow cytometry analysis. ELISA was used to measure the levels of tumour necrosis factor (TNF)‐ɑ, IL‐1β, IL‐6, IL‐8 and MCP‐1. The results showed that honokiol (0.5–20 μM) showed non‐toxic effects on BEAS‐2B cells. Treatment with honokiol (2, 5 and 10 μM) reduced CSE (300 mg/L)‐induced decrease in cell viability and apoptosis in BEAS‐2B cells. Honokiol also decreased CSE‐induced inflammation through inhibiting expression and secretion of inflammatory cytokines, such as TNF‐ɑ, IL‐1β, IL‐6, IL‐8 and MCP‐1. Moreover, honokiol repressed CSE‐induced reactive oxygen species (ROS) production, decrease of ATP content and mitochondrial biogenesis, as well as mitochondrial membrane potential. Mechanistically, honokiol promoted the expression of SIRT3 and its downstream target genes, which are critical regulators of mitochondrial function and oxidative stress. Silencing of SIRT3 reversed the protective effects of honokiol on CSE‐induced damage and mitochondrial dysfunction in BEAS‐2B cells. These results indicated that honokiol attenuated CSE‐induced damage of airway epithelial cells through regulating SIRT3/SOD2 signalling pathway.

## 1 INTRODUCTION

COPD is a type of progressive lung disease with high morbidity and mortality, which is characterized by long‐term airflow limitation and respiratory symptoms such as shortness of breath and a cough. Although COPD is incurable, it is treatable and preventable. Recent studies show that COPD can make people more likely to get severely ill when infected with COVID‐19.^1^ Although exposure to air pollutants, respiratory infections and genetic factors play critical roles in the progression of COPD, cigarette smoking is considered to be the main risk factor of COPD in Western countries and China.^2^, ^3^ It has been reported that cigarette smoking‐induced abnormal inflammatory responses contributes to COPD.^3^ Neutrophilic inflammation mediated by IL‐1ɑ is observed to be increased in patients with COPD.^4^ IL‐1β is also found to be induced in cigarette smoke extract (CSE)‐induced airway epithelial cells.^5^ Cigarette smoke can cause inflammation and induce apoptosis of alveolar epithelial cells via release of TNF‐ɑ.^6^ Macrophages within the lungs can produce IL‐8/CXCLs, leading to increased inflammatory response through inducing leukocytes from circulatory systems to the inflammatory site.^7^ In addition, other pro‐inflammatory cytokines (IL‐1β and IL‐6) are also proved to be critical mediators in CSE‐induced lung inflammation in COPD.^8^

Oxidative stress plays crucial roles in a variety of disorders including COPD. Cigarette smoke exposure results in increased oxidative stress in bronchial epithelial cells due to unbalance between oxidants and antioxidants.^9^, ^10^ Meanwhile cigarette smoke indirectly increases oxidative stress through inhibiting the activity of major endogenous antioxidant genes, such as nuclear factor erythroid 2‐related factor 2 (Nrf2) and superoxide dismutase 2 (SOD2).^11^ Mitochondrial are complex organelles that play central roles in cellular energy metabolism and ROS generation. It has been reported that mitochondrial dysfunction contributes to the pathogenesis of COPD.^12^, ^13^, ^14^, ^15^, ^16^ Decreased oxidative stress and inflammation contributes to improved respiratory dysfunction in COPD rats.^17^

Sirtuins are NAD + ‐dependent protein deacetylases. The activities of sirtuins are regulated in response to multiple external stimulation and stress responses, such as drug exposure, glucose deprivation and metabolic stress.^18^, ^19^, ^20^ SIRT3 is reported to be localized in the mitochondrial matrix and promotes deacetylation of multiple metabolic enzymes in response to metabolic changes.^21^ Hyperacetylation of several mitochondrial proteins are observed in SIRT3^−/−^ mice.^20^ SIRT3 controls mitochondrial oxidative pathways and influences mitochondrial ROS production.^22^ Interestingly, SIRT3 promotes the expression of PGC‐1ɑ to protect cells against oxygen–glucose deprivation‐induced neuronal death.^23^ Recently, SIRT3 has been found to be involved in cigarette smoke‐induced COPD. SIRT3 decreases airway epithelial mitochondrial oxidative stress in CSE‐treated human bronchial epithelial cells and COPD rat model.^16^

Honokiol isolated from Magnolia species is a phenolic compound with multiple biological activities, including antitumor, antimicrobial, hepatoprotective and cardioprotective effects.^24^ Recent studies show that honokiol can decrease β‐secretase activity leading to reduced amyloid beta levels through upregulating the expression of PGC‐1ɑ and SIRT3, suggesting that honokiol has neuroprotective property.^25^ Honokiol was reported to induce apoptosis of non‐small cell lung cancer (NSCLC) cells and inhibit NSCLC cells migration via different mechanisms.^26^, ^27^, ^28^ However, whether honokiol has protective property against CSE‐induced injury in bronchial epithelial cells remains unclear.

In the present study, we investigated the protective effects of honokiol on CSE‐induced oxidative stress, apoptosis, inflammation, and mitochondrial dysfunction in BEAS‐2B cells. We also explored the underlying molecular mechanism.

## 2 MATERIALS AND METHODS

### 2.1 Chemicals and reagents

Primary antibodies against β‐actin (WL01372), SIRT3 (WL03840), SOD1 (WL01846), SOD2 (WL03840) and NRF2 (WL02135) were purchased from Wanleibio Technology Inc. (Shenyang, China). Cell Counting Kit‐8 (CCK‐8) and the LDH activity assay kit were obtained from Beyotime Institute of Biotechnology (Hangzhou, Zhejiang, China). Bicinchoninic acid (BCA) protein assay kit was obtained from Thermo Fisher Scientific (San Diego, CA, USA). Dimethylsulfoxide (DMSO) was from Sigma‐Aldrich (St. Louis, MO, USA). FBS was obtained from Gibco (Thornton, NSW, Australia). Honokiol (≥98%, H111272) was purchased from Aladdin (Shanghai, China).

### 2.2 Cell culture and treatment

BEAS‐2B cells were obtained from Procell (Wuhan, China) and maintained in M199 medium containing 10% FBS at 37°C with 5% CO_2_ and 95% air, 100 U/mL penicillin and 100 μg/mL streptomycin sulfate. The medium was replenished every 2 days.

CSE was prepared as described previously and stored at −80°C.^7^ Briefly, a waterpipe smoking device was designed, and the smoke was allowed to flow into a plastic bottle submerged in liquid nitrogen. The cigarettes Daqianmen containing 11 mg tar and 0.8 mg nicotine were used. The condensate in the wall of plastic bottle was collected, weighed, dissolved in DMSO at a concentration of 400 mg/mL, and then stored at −80°C.

### 2.3 Cell viability assay

BEAS‐2B cells were seeded into a 96‐well plate (1.5 × 10^4^ cells per well). After washed with PBS for three times, cells were maintained in fresh medium with different concentrations of CSE in the absence/presence of honokiol for another 24 h. Cell viability was analysed by a CCK‐8 kit.

### 2.4 LDH leakage assay

Cells were incubated with CSE with or without honokiol for indicated time, then the LDH activity in the supernatant was measured using a LDH assay kit according to the manufacturer's instructions.

### 2.5 Real‐time quantitative PCR

Total RNA was extracted from BEAS‐2B cells using TRIzol Reagent (Ambion, Austin, TX, USA), and cDNA was synthesized using the cDNA reverse transcriptase kit (TOYOBO Biotech, Osaka, Japan). Real‐time PCR was performed on a Bio‐Rad CFX Connect platform. β‐actin was used as an internal control. Relative mRNA levels were analysed using the 2^−ΔΔCt^ method.

### 2.6 Western blotting analysis

Total protein was prepared from BEAS‐2B cells by RIPA buffer (Beyotime Bio, Shanghai, China). Protein concentrations were measured by a BCA protein assay kit. The protein was separated by SDS‐PAGE, subsequently transferred to a polyvinylidene difluoride membrane. After blocked with 3% BSA, the membranes were then immunoblotted with different primary antibodies (SIRT3, SOD1, SOD2 and β‐actin, 1:1000) for about 12 h. Then membranes were washed five times for 10 min with TBST and incubated with HRP‐conjugated secondary antibody (1:8000, WLA023, Wanlei Bio or 1:5000, WLA024, Wanlei Bio) for 1 h at room temperature. Subsequently, the membranes were washed for additional five times and were visualized by an enhanced chemiluminescence detection kit.

### 2.7 Enzyme‐linked immunosorbent assay (ELISA)

The levels of TNF‐ɑ, IL‐1β, IL‐6, IL‐8 and MCP‐1 in the supernatant were determined by commercial ELISA kits (Beyotime Institute of Biotechnology, Hangzhou, Zhejiang, China).

### 2.8 Flow cytometry analysis of BEAS‐2B cells apoptosis

BEAS‐2B cells were seeded in six‐well plates and treated as indicated for 24 h. Cells were collected by trypsinization and resuspended in 300 μL of binding buffer. Afterward, cells were incubated with 5 μL of annexin V‐FITC and 5 μL of propidium iodide. Cells were incubated on ice for 30 min after washed for additional two times. Finally, cells were analysed by a flow cytometer (FACSCelesta; BD Biosciences, Franklin Lakes, NJ, USA).

### 2.9 Measurement of ROS production

ROS levels were measured by a DCFH‐DA kit. BEAS‐2B cells were cultured in six‐well plates and treated with CSE for 4 h. Cells were then washed twice with PBS buffer, subsequently incubated with 10 μM of DCFH‐DA for 30 min at 37°C in the dark. Then cells were collected and the fluorescent intensity was determined by a multimode microplate reader (Berthold TriStar LB941, Germany). Data were normalized to the corresponding total protein.

### 2.10 Measurement of mitochondrial membrane potential (MMP)

MMP was analysed by a JC‐1 assay kit. Briefly, cells were first incubated with JC‐1 dye in the dark at 37°C for 20 min and then rinsed with washing buffer. Finally, fluorescence images of BEAS‐2B cells were analysed by a fluorescence microscope (Leica DMi8, Germany).

### 2.11 Measurement of ATP production in BEAS‐2B cells

The ATP content was measured using an ATP assay kit. Briefly, cells were lysed on ice, then centrifuged at 12000 × *g* for 5 min. 90 μL of each supernatant was added to 100 μL of working solution. Luminescence was measured using a microplate reader (Berthold TriStar LB941, Germany). ATP levels were normalized to total protein contents of each sample.

### 2.12 Measurement of mitochondrial DNA (mtDNA) copy number

Total DNA was isolated from BEAS‐2B cells using a DNA isolation kit (Tiangen Biotech, Beijing, China). The DNA content of each sample was adjusted to be consistent. Real‐time PCR was carried out and the data were analysed by the 2^−ΔΔCT^ method.

### 2.13 RNA sequencing

BEAS‐2B cells were treated with DMSO, CSE, CSE + HNK (5 μM), then samples were collected and sent to Wuhan Metware Biotechnology Co., Ltd. (Wuhan, China) for RNA‐seq analysis by Illumina sequencing platforms. Differentially expressed genes were identified for GO and KEGG pathway enrichment analysis.

### 2.14 Statistical analysis

The data were expressed as mean ± standard deviation (SD) and analysed using GraphPad Prism 7 (GraphPad Software, CA, USA). The significance was evaluated using one‐way analysis of variance (anova) followed by Tukey's multiple comparisons (multiple groups). An unpaired Student's *t*‐test was used to analyse the statistical significance between two groups. *p* value <0.05 was considered statistically significant.

## 3 RESULTS

### 3.1 Effects of honokiol on CSE‐induced apoptosis in BEAS‐2B cells

**FIGURE 1:**
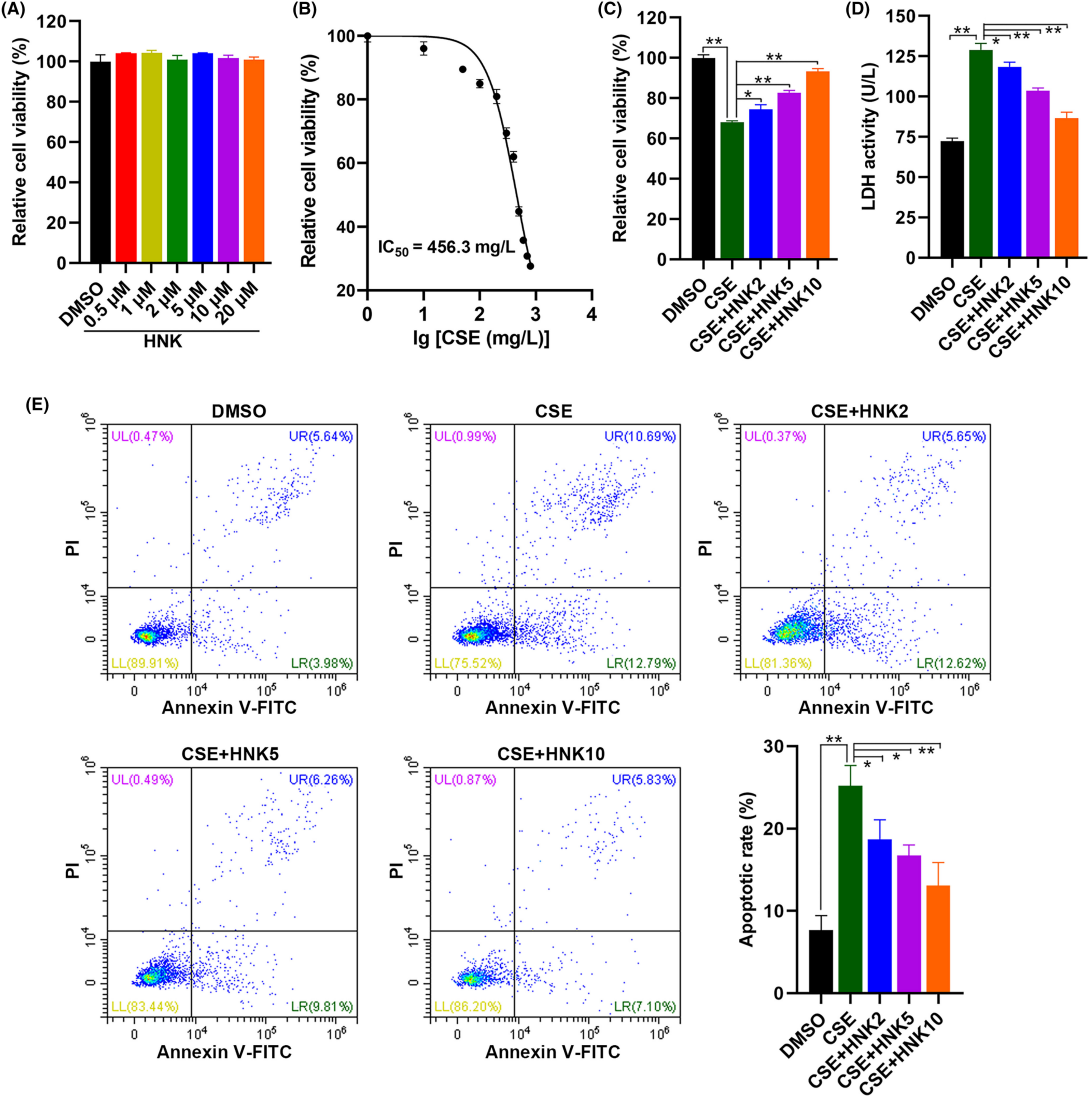
Protective effects of honokiol on viability of BEAS‐2B cells exposed to CSE. Cells were treated with honokiol (A), CSE (B) or honokiol + CSE (C), then cell viability was analysed. Cells were treated as in (C), then LDH leakage (D) and apoptosis (E) were analysed. The data are represented as mean ± SD; *p < 0.05, **p < 0.01.

To investigate the protective effects of honokiol on CSE‐induced BEAS‐2B cell injury, cells were firstly treated with honokiol (0.5–20 μM) for 24 h, and the cell viability was measured using an MTT assay. No toxic effect was observed after treatment with honokiol from 0.5 to 20 μM for 24 h (Figure 1A). While incubated with CSE (50–800 mg/L) caused obvious decreases in cell viability (Figure 1B), and the IC_50_ value was 456.3 mg/L. Further treatment with honokiol (2–10 μM) significantly improved cell viability as shown in Figure 1C. Incubation with CSE also markedly increased the release of lactate dehydrogenase from BEAS‐2B cells, whereas this effect was reversed after further treatment with honokiol (Figure 1D). To determine whether honokiol showed a protective role in CSE‐induced apoptosis in BEAS‐2B cells, a flow cytometric assay was employed. As shown in Figure 1E, BEAS‐2B cell apoptosis was dramatically increased after administration of CSE, while as expected, combined treatment with honokiol attenuated the increased apoptosis rate caused by CSE exposure. These results strongly suggest that honokiol displays protective effects against CSE‐induced injury of bronchial epithelial cell.

### 3.2 Effects of honokiol on CSE‐induced expression and secretion of inflammatory cytokines in BEAS‐2B cells

**FIGURE 2:**
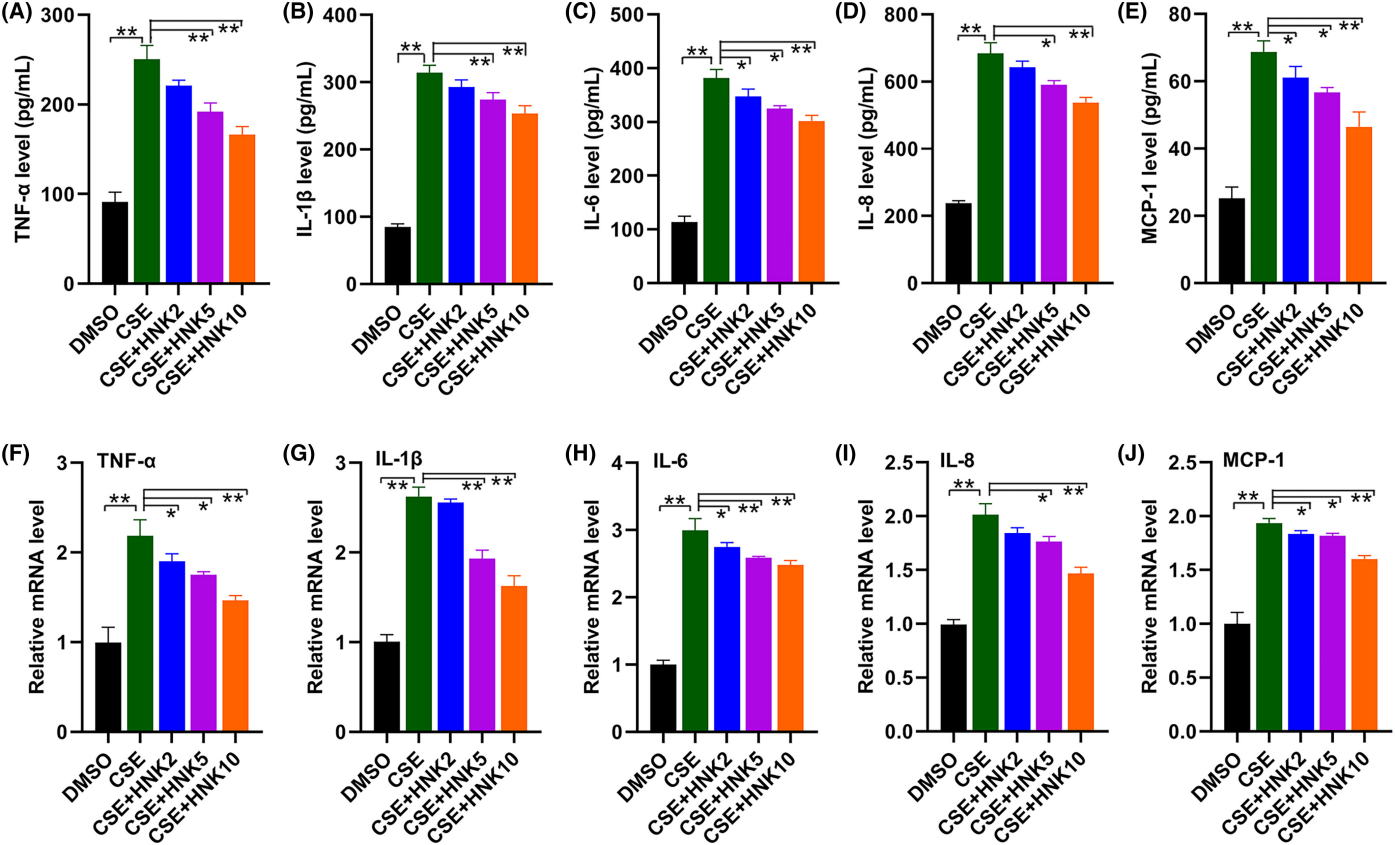
Protective effects of honokiol on release of inflammatory cytokines in BEAS‐2B cells exposure to CSE. Cells were treated with CSE in the presence/absence of honokiol, the protein and mRNA levels of TNF‐ɑ (A, F), IL‐1β (B, G), IL‐6 (C, H), IL‐8 (D, I) and MCP‐1 (E, J) were determined by ELISA and RT‐qPCR, respectively. The data are represented as mean ± SD; *p < 0.05, **p < 0.01.

To investigate whether the CSE‐induced inflammatory response was regulated by honokiol, an enzyme‐linked immunosorbent assay was used to determine the inflammatory cytokines in the cell supernatants. Exposure to CSE increased the release of TNF‐ɑ, IL‐1β, IL‐6, IL‐8 and MCP‐1 (Figure 2A–E), while pretreatment with honokiol significantly lowered the expression of these cytokines in the cell supernatant. We further examined the effects of honokiol on mRNA level of these genes. RT‐qPCR data indicated that mRNA expression of TNF‐ɑ, IL‐1β, IL‐6, IL‐8 and MCP‐1 were significantly induced after stimulation of BEAS‐2B cells with CSE, which were reduced by further treatment with honokiol (Figure 2F–J). These data indicate that improvement of inflammatory response may play critical roles in protecting BEAS‐2B cells against CSE‐induced injury.

### 3.3 Effects of honokiol on CSE‐induced oxidative stress, mitochondrial DNA content and mitochondrial dysfunction in BEAS‐2B cells

**FIGURE 3:**
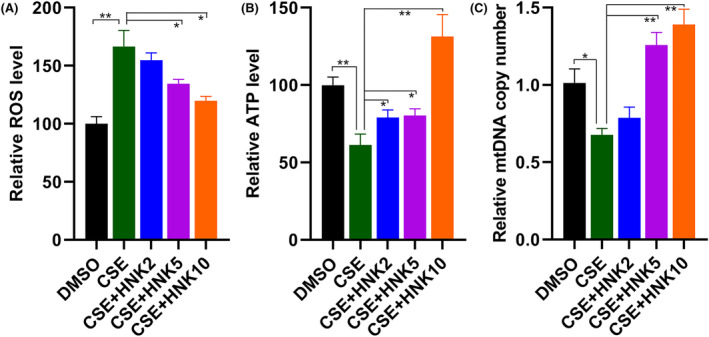
Protective effects of honokiol on ROS generation and mitochondrial dysfunction in BEAS‐2B cells exposure to CSE. Cells were treated with CSE in the presence/absence of honokiol, and the ROS production (A), ATP levels (B) and mitochondrial DNA content (C) were determined. The data are represented as mean ± SD; *p < 0.05, **p < 0.01.

To determine whether honokiol can attenuate CSE exposure caused oxidative stress, the ROS levels in BEAS‐2B cells were analysed after treated with CSE. As shown in Figure 3A, treatment with CSE significantly increased ROS levels, which were decreased by honokiol treatment. We next assessed the intracellular ATP levels and found that exposure to CSE markedly led to decreased ATP content, suggesting impaired cellular energy metabolism (Figure 3B). By contrast, further treatment with honokiol dramatically restored the ATP levels. Interestingly, the ATP levels in high dose of honokiol‐treated group were much higher than that treated with DMSO control group (Figure 3B).

**FIGURE 4:**
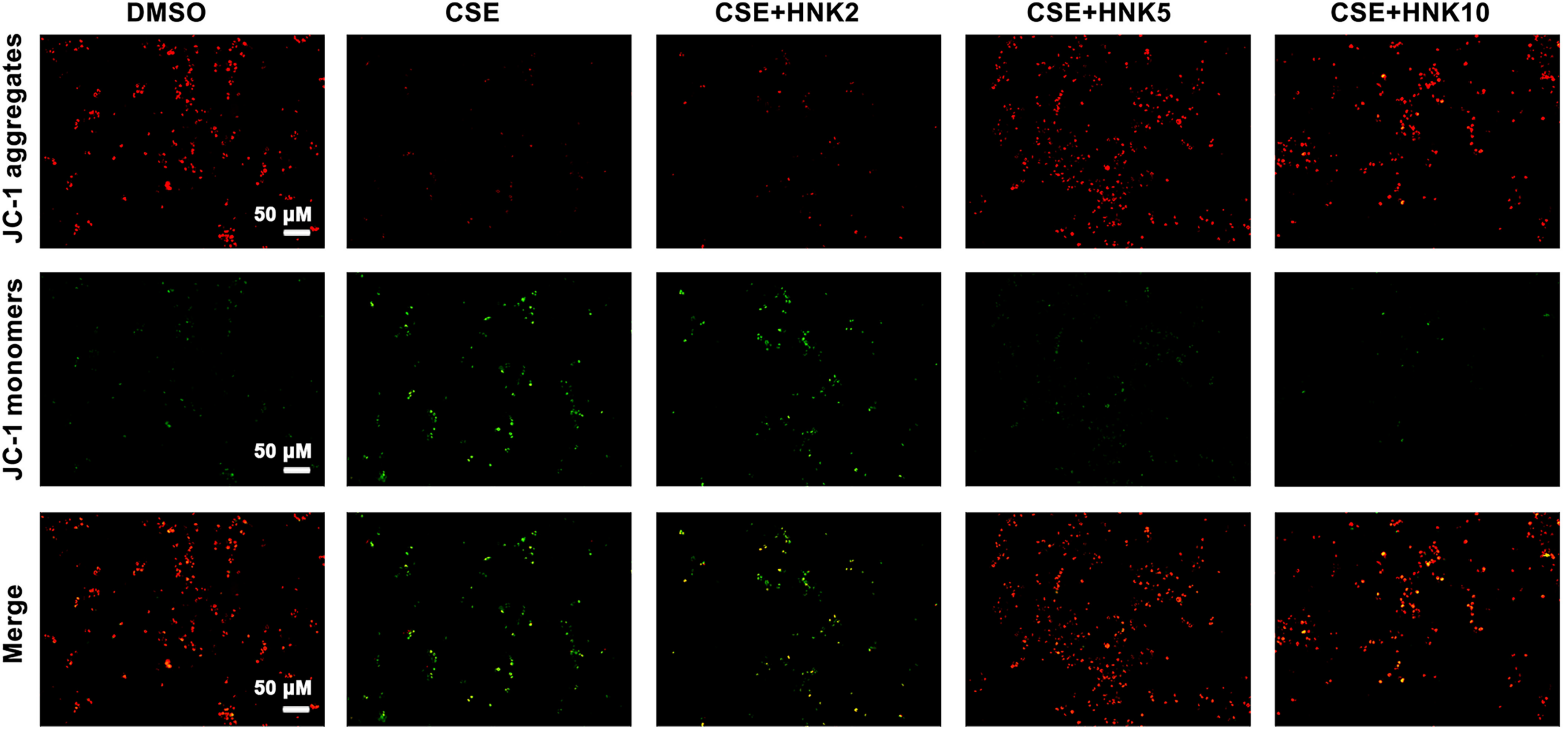
Protective effects of honokiol on MMP in BEAS‐2B cells exposure to CSE. Cells were treated with CSE in the presence/absence of honokiol, MMP was analysed using a JC‐1 assay kit.

Given the fact that mitochondrial plays important roles in ROS and ATP production, we further analysed the effects of CSE and honokiol on mitochondrial biogenesis. The result from quantitative analysis of mitochondrial DNA content indicated that CSE exposure inhibited the mitochondrial biogenesis, in contrast, the inhibitory effect was reversed by further treatment with medium‐ or high‐doses of honokiol, although low‐dose of honokiol exposure had no effect on mitochondrial DNA copy number (Figure 3C), which was restored to near or even higher than control group when exposure to high‐dose of honokiol. We next evaluated the effects of honokiol on mitochondrial membrane potential. As shown in Figure 4, the result indicated that the mitochondrial membrane potential was markedly decreased after CSE treatment, as evidenced by the increased green but decreased red fluorescence in BEAS‐2B cells. Moreover, cotreatment with honokiol elevated mitochondrial membrane potential, suggesting improved mitochondrial function.

### 3.4 Effects of honokiol on SIRT3 signalling pathway

**FIGURE 5:**
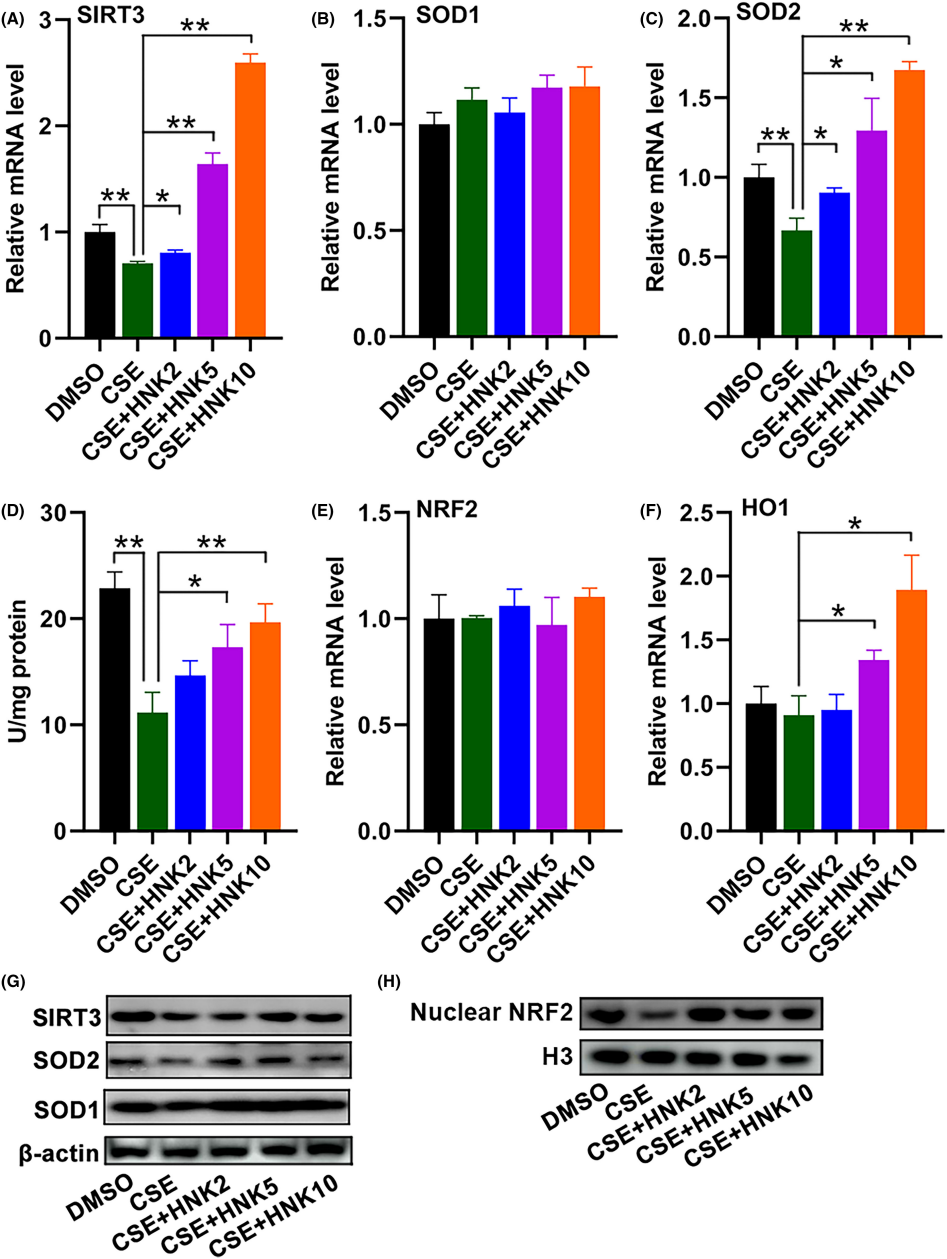
Honokiol attenuated CSE‐induced damage of BEAS‐2B cells through regulation of SIRT3/SOD2 axis. Cells were treated with CSE in the presence/absence of honokiol, and the mRNA levels of SIRT3 (A), SOD1 (B), SOD2 (C), NRF2 (E) and HO1 (F) were analysed by RT‐qPCR. (D) Cells were treated as in (A), the enzymatic activity of SOD2 was measured by a SOD2 assay kit. The protein expression of SIRT3, SOD2, SOD1 (G) and nuclear NRF2 (H) were analysed by western blotting. The data are represented as mean ± SD; *p < 0.05, **p < 0.01.

SIRT3 was found to be located in mitochondrial, and dysregulation of SIRT3 contributed to impaired mitochondrial function. We therefore investigated whether SIRT3 was regulated by honokiol in CSE‐treated BEAS‐2B cells. The result suggested that exposure to CSE significantly decreased the mRNA expression of SIRT3 (Figure 5A) and SOD2 (Figure 5C), while no changes in mRNA levels of SOD1 were observed (Figure 5B). Further incubation with honokiol led to an obvious increase in mRNA levels of these genes. In line with the increased mRNA expression, we also found that CSE treatment resulted in a significant increase in the enzyme activity of SOD2 in CSE‐treated BEAS‐2B cells (Figure 5D).

Previous studies have revealed that NRF2/HO1 axis also plays crucial roles in regulation of oxidative stress in bronchial epithelial cells, then we next studied whether this signalling pathway was affected by honokiol. The results demonstrated that treatment with CSE has no effect on mRNA levels of NRF2 or HO1 (Figure 5E, F), while honokiol treatment significantly promoted the mRNA expression of HO1 by about 1.8 fold (Figure 5F). The protein expression of SIRT3, SOD1/2 and nuclear NRF2 were significantly decreased in CSE‐treated BEAS‐2B cells (Figure 5G, H), which were at least partially restored by further treatment with honokiol, consistent with the results from mRNA level and enzyme activity assay. These results indicated that SIRT3 signalling pathway might contribute to the protective effects of honokiol against CSE‐induced injury of bronchial epithelial cells.

### 3.5 SIRT3 mediated the protective effects of honokiol in CSE‐induced bronchial epithelial cells

**FIGURE 6:**
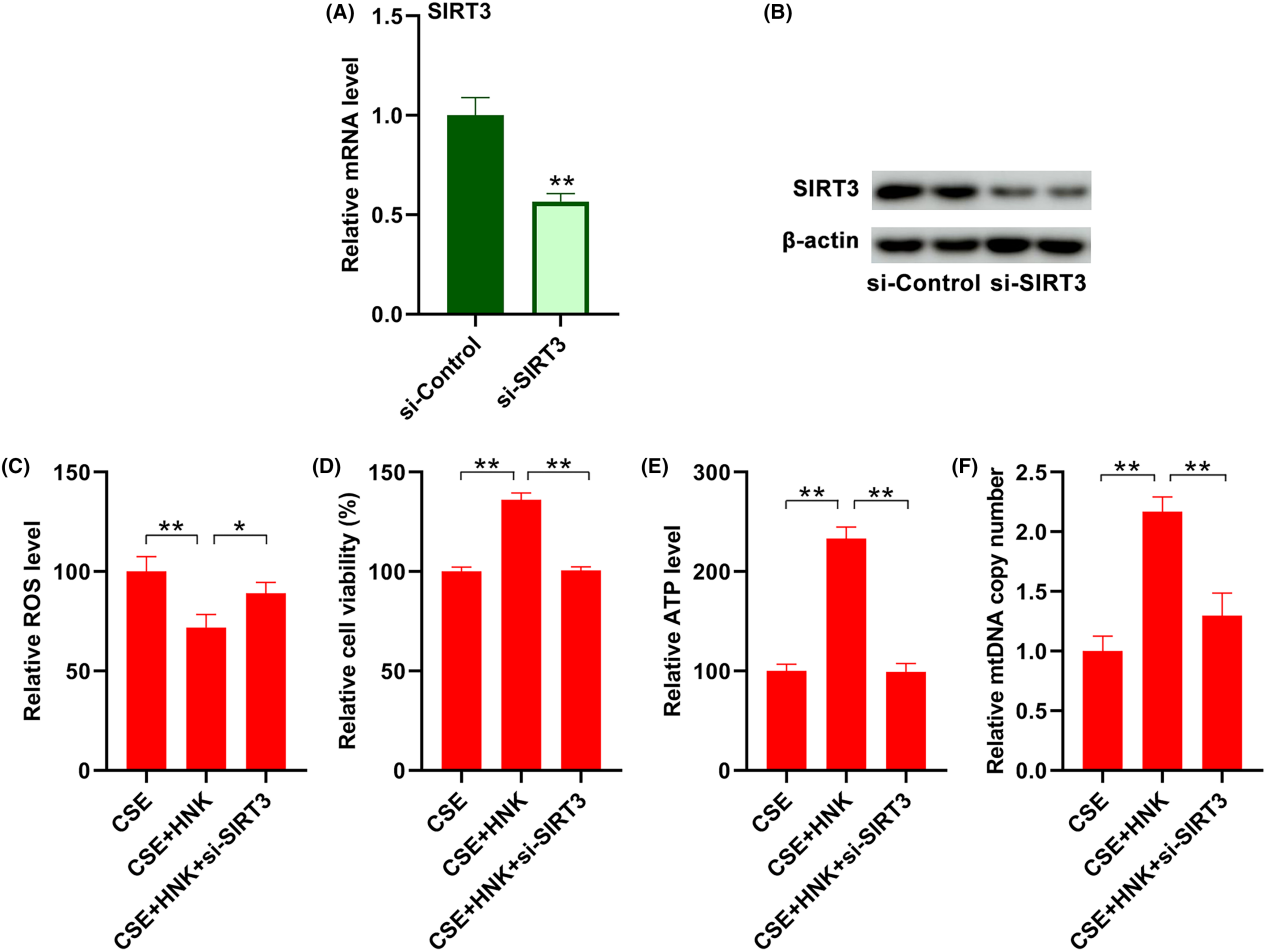
SIRT3 mediated the effects of honokiol on CSE‐induced injury in BEAS‐2B cells. Cells were transfected with control siRNA or SIRT3 siRNA, then the mRNA (A) and protein (B) levels of SIRT3 were analysed by RT‐qPCR and Western blotting. Cells were treated with CSE and honokiol in the presence/absence of SIRT3 siRNA, and ROS (C), cell viability (D), ATP levels (E) and mitochondrial DNA content (F) were measured. The data are represented as mean ± SD; *p < 0.05, **p < 0.01.

To further confirm that SIRT3 mediated the improvement of CSE‐induced oxidative stress and mitochondrial damage, siRNA targeting SIRT3 (si‐SIRT3) and siRNA targeting Control (si‐Control) was employed. As shown in Figure 6A, transfection of si‐SIRT3 resulted in significant decrease in SIRT3 mRNA levels, as evidenced by a RT‐qPCR assay. Accordingly, we also observed obvious downregulation of SIRT3 protein levels after treatment with si‐SIRT3 as compared with si‐Control (Figure 6B). Consistently, silencing of SIRT3 significantly increased ROS levels in CSE‐treated BEAS‐2B cells (Figure 6C). In addition, downregulation of SIRT3 at least partially reversed the inductive effects on cell viability (Figure 6D), ATP level (Figure 6E), as well as mitochondrial DNA copy number (Figure 6F) in CST‐treated BEAS‐2B cells. The results suggested that SIRT3‐mediated mitochondrial function recovery, at least in part, contributed to the protective effects of honokiol on CSE‐induced injury of BEAS‐2B cells.

### 3.6 Effects of honokiol on transcriptome in BEAS‐2B cells

**FIGURE 7:**
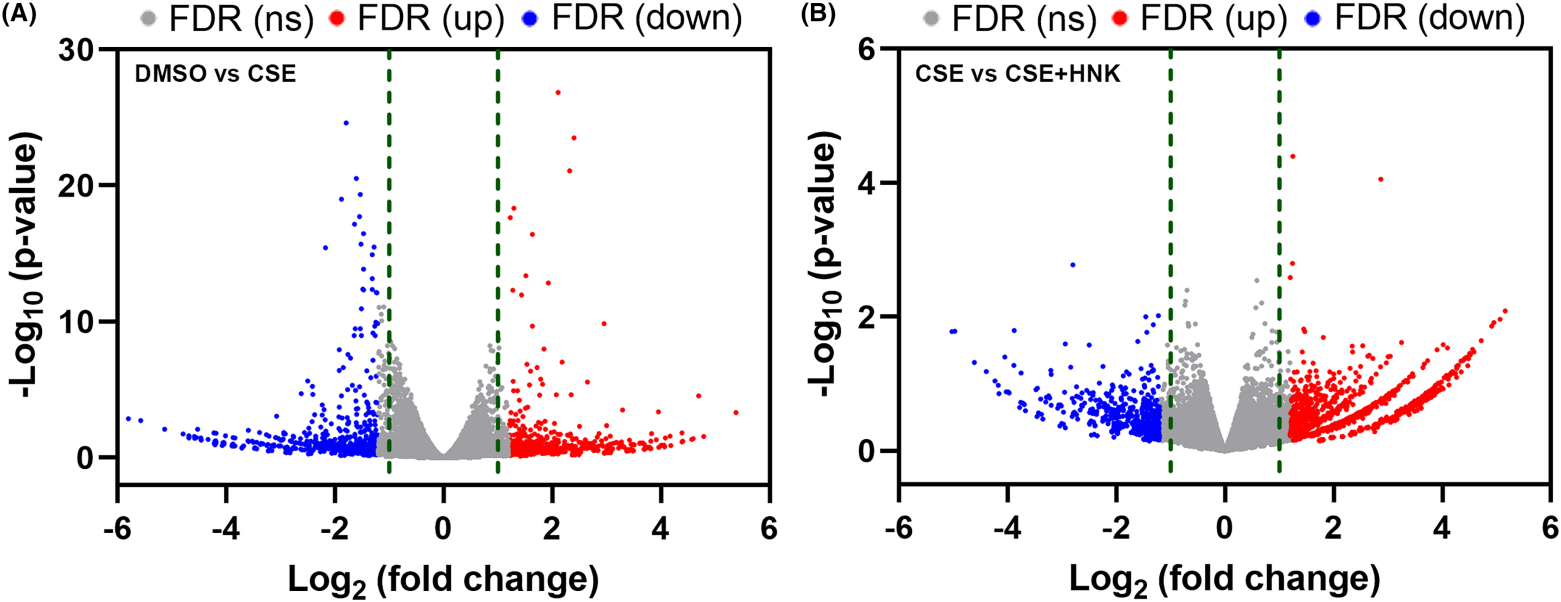
Volcano plots representing the *p*‐values and the log_2_ fold change values (cutoff ≥2.0) of genes in DMSO groups versus CSE‐treated groups (A) or CSE groups versus CSE + HNK groups (B).

To further reveal the molecular pathway by which honokiol regulates CSE‐induced airway epithelial cells injury, RNA sequencing (RNA‐seq) was performed. As shown in Figure 7, volcano plots represented the *p*‐values and the log_2_ fold change values (cutoff ≥2.0) of genes in DMSO groups versus CSE‐treated groups (Figure 7A) or in CSE‐treated groups versus CSE/honokiol‐treated groups (Figure 7B).

**FIGURE 8:**
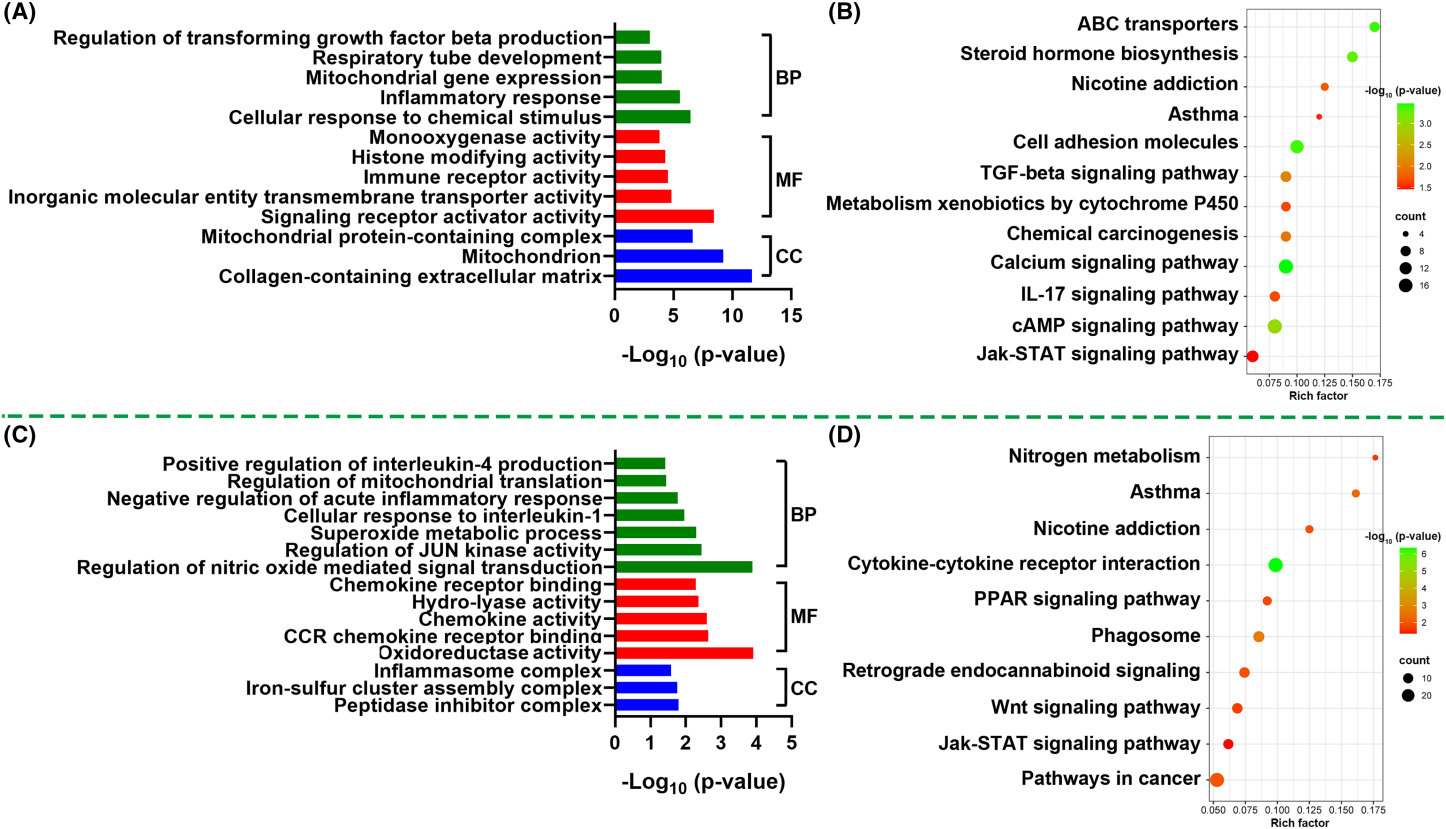
Comparative analysis of the enrichment of differentially expressed genes in GO and KEGG pathways following treatment with DMSO versus CSE (A, B) or treatment with CSE versus CSE + HNK (C, D) in BEAS‐2B cells, respectively. BP, biological process; MF, molecular function; CC, cellular component.

Moreover, the results from Gene Ontology (GO) and Kyoto Encyclopedia of Genes and Genomes (KEGG) enrichment analysis indicated that TGF‐β signalling pathway, inflammatory response, mitochondrial gene expression, cellular response to chemical stimulus, collagen‐containing extracellular matrix and Jak–STAT signalling pathway were markedly impaired by CSE treatment as compared with DMSO treatment (Figure 8A, B). While, IL‐1 and IL‐4 pathway, negative regulation of acute inflammatory response, regulation of mitochondrial translation, superoxide metabolic process, nitric oxide‐mediated signal transduction and nicotine addiction were enriched after treatment with CSE + HNK as compared with CSE treatment alone (Figure 8C, D).

## 4 DISCUSSION

Cigarette smoking is considered to be the major risk factor for COPD.^29^ Cigarette smoke alters normal airway epithelial barrier function and induces bronchial epithelial cell apoptosis in vitro^30^, ^31^ and in vivo.^32^ We found that CSE exposure decreased the viability but increased apoptosis of BEAS‐2B cells, which is consistent with previous reports. Further treatment with honokiol reversed this effect. Indeed, several chemicals, such as bronchodilators tiotropium and olodaterol, have been found to inhibit CSE‐induced apoptosis of BEAS‐2B cells though upregulation of phosphorylation and activation of JNK. Interestingly, they showed that CSE treatment also resulted in autophagy, another form of cell death, in BEAS‐2B cells.^33^ Whether treatment with honokiol can inhibit CSE‐induced autophagy need to be further investigated in the future.

Previous studies have reported that inflammatory cytokines levels (TNF‐ɑ, MCP‐1 and IL‐8) are higher in COPD patients than that in control subjects.^34^, ^35^ Meanwhile, these inflammatory mediators (TNF‐ɑ, IL‐8 and IL‐6) were also found to be increased in CSE‐treated RAW264.7 and bronchial epithelial cells,^34^, ^36^ suggesting that cigarette smoke exposure results in chronic inflammation subsequently leading to progressive airflow limitation in COPD. Our results clearly demonstrated that the mRNA and protein levels of TNF‐ɑ, IL‐β, IL‐6, IL‐8 and MCP‐1 were upregulated in response to CSE treatment, which were reversed by further treatment with honokiol, suggesting reduced inflammatory response. Recently, phloretin and Theaflavin‐3,3′‐digallate were reported to improve airway inflammation caused by cigarette smoking in inbronchial epithelial cells and mouse lung through inhibiting the expression of TNF‐ɑ and IL‐1β,^37^, ^38^ which is consistent with our results. Moreover, Costa A and coauthors reported that honokiol protected skin cells against cigarette smoke‐induced inflammation and apoptosis.^39^ Hong T et al also revealed that oral administration of honokiol attenuated airway inflammation in an ovalbumin‐induced asthmatic mouse model.^40^

It was reported that cigarette smoking could lead to ROS and RNS production, which were considered to contribute to the development of COPD.^41^, ^42^ Moreover, the increased mitochondrial ROS generation and decreased mitochondrial ATP production were supposed to play crucial roles in the pathophysiology of COPD. These data suggested that mitochondrial function was closely related with CSE‐induced COPD. The present study showed that CSE exposure significantly increased ROS generation but decreased ATP production, which were attenuated after honokiol treatment, suggesting that honokiol may show protective ability against CSE‐induced mitochondiral dysfunction. To test this hypothesis, BEAS‐2B cells were treated with honokiol in the presence of CSE, and we found that incubation with honokiol significantly improved CSE exposure‐induced decrease in mitochondrial biogenesis and mitochondrial membrane potential, which was consistent with previous study showing that honokiol treatment attenuated deterioration of mitochondrial function in chemicals‐induced acute kidney injury and liver damage.^43^, ^44^, ^45^, ^46^ However, another study showed that treatment with honokiol resulted in promoted mitochondrial swelling, decreased membrane potential and affected respiration of mitochondrial, indicating impaired mitochondrial function induced by honokiol.^47^ These contradictory data might be explained by the fact that much higher concentrations of honokiol (25–100 μM) were used to directly treat mitochondrial isolated from the rat liver. Thus, these data suggest that low concentrations of honokiol may show a protective role in CSE‐induced COPD, while high concentrations of honokiol may cause opposite effects. Since multiple effects of honokiol on different tissues and diseases were observed,^48^ the effect of honokiol on lung epithelial cells in vivo was still needed to be investigated.

SIRT3 and NRF2 pathways were considered to be important regulators of mitochondrial function, including ROS and ATP production, lipid acid oxidation and oxidative phosphorylation.^23^ To further explore the molecular mechanism mediating the protective effects of honokiol on CSE‐induced damage of BEAS‐2B cells, we focused on the SIRT3/SOD2 and NRF2/HO1 axis. The data from Western blotting indicated that the nuclear accumulation of NRF2 protein was increased by honokiol and CSE treatment as compared with CSE treatment alone. Previous studies have suggested that the transcriptional activity of NRF2 was regulated by its nuclear translocation.^49^, ^50^, ^51^ Furthermore, the results showed that honokiol treatment significantly improved the inhibitory effects of CSE on mRNA expression of SIRT3 and SOD2 with no affection on mRNA expression of SOD1, indicating that SOD2 was the main target gene of SIRT3 in BEAS‐2B cells. More importantly, the reduced activity of SOD2 caused by exposure to CSE was recovered when cells were treated with honokiol. Similar results were observed on protein expression of SIRT3 and SOD2. Since the deacetylation of SOD2 can be directly regulated by SIRT3, detection of SOD2 acetylation level in BEAS‐2B cells treated by honokiol needs to be further investigated. However, treatment with CSE showed no impairment on mRNA levels of NRF2 and HO1, while 5 and 10 μM of honokiol significantly increased mRNA level of HO1. Taken together, these data indicated that SIRT3/SOD2 signalling pathway was involved in the regulation of mitochondrial function in CSE‐treated BEAS‐2B cells.

Finally, siRNA oligonucleotides targeting SIRT3 (si‐SIRT3) were used to further confirmed that SIRT3 mediated the protective effects of honokiol on CSE‐induced injury in BEAS‐2B cells. Interestingly, several phenotypes such as ROS levels and mitochondrial copy number were partially reversed after treatment with si‐SIRT3. Two possible explanations are as follows: (1) mRNA and protein expression were not fully inhibited by si‐SIRT3 treatment. (2) We cannot rule out the possibility that other sirtuins such as SIRT1 may also be regulated by honokiol. Therefore, whether honokiol can still protect against CSE‐induced injury in SIRT3 knockout BEAS‐2B cells remains to be investigated in the future.

There are also several limitations in the present study: (1) Whether similar effects of honokiol on other airway epithelial cells (such as 16HBE) could be observed still need further investigation. (2) Animal models are required to confirm whether honokiol still show protective effects on airway epithelial cells in vivo. (3) Since mild and restricted activation of inflammatory response is beneficial for the recovery of CSE‐induced injury of airway epithelial cells, the effects of honokiol on immune cells need to be further investigated.

## 5 CONCLUSION

In summary, our results demonstrate that honokiol can protect bronchial epithelial cells against CSE‐induced damage though SIRT3/SOD2 pathway.

## AUTHOR CONTRIBUTIONS

**Fei Li:** Conceptualization (lead); funding acquisition (lead); project administration (lead); supervision (lead); writing – original draft (lead). **Chunyu Ye:** Data curation (equal). **Xiuli Wang:** Data curation (equal); formal analysis (equal); methodology (equal). **Xinting Li:** Data curation (equal); investigation (equal); methodology (equal). **Xiaoxia Wang:** Supervision (equal); writing – review and editing (equal).

## FUNDING INFORMATION

This study was supported by Applied Basic Research Program, the Natural Science Foundation for Young Scientists of Shanxi Province, China (No. 201701D221264) and Ph.D. Research Fund of Shanxi Provincial People's Hospital (No. b201615).

## CONFLICT OF INTEREST STATEMENT

The authors declare that they have no known competing financial interests or personal relationships that could have appeared to influence the work reported in this paper.

## Data Availability

Data are available from the corresponding author on reasonable request.

## References

[1] Song WJ , Hui C , Hull JH , et al. Confronting COVID‐19‐associated cough and the post‐COVID syndrome: role of viral neurotropism, neuroinflammation, and neuroimmune responses. Lancet Respir Med. 2021;9(5):533‐544. 33857435 10.1016/S2213-2600(21)00125-9 PMC8041436

[2] Wang C , Xu J , Yang L , et al. Prevalence and risk factors of chronic obstructive pulmonary disease in China (the China pulmonary health [CPH] study): a national cross‐sectional study. Lancet. 2018;391(10131):1706‐1717. 29650248 10.1016/S0140-6736(18)30841-9

[3] Mannino DM , Buist AS . Global burden of COPD: risk factors, prevalence, and future trends. Lancet. 2007;370(9589):765‐773. 17765526 10.1016/S0140-6736(07)61380-4

[4] Pauwels NS , Bracke KR , Dupont LL , et al. Role of IL‐1α and the Nlrp3/caspase‐1/IL‐1β axis in cigarette smoke‐induced pulmonary inflammation and COPD. Eur Respir J. 2011;38(5):1019‐1028. 21622588 10.1183/09031936.00158110

[5] Dang X , He B , Ning Q , et al. Alantolactone suppresses inflammation, apoptosis and oxidative stress in cigarette smoke‐induced human bronchial epithelial cells through activation of Nrf2/HO‐1 and inhibition of the NF‐κB pathways. Respir Res. 2020;21(1):95. 32321531 10.1186/s12931-020-01358-4 PMC7178609

[6] Guan R , Wang J , Li D , et al. Hydrogen sulfide inhibits cigarette smoke‐induced inflammation and injury in alveolar epithelial cells by suppressing PHD2/HIF‐1α/MAPK signaling pathway. Int Immunopharmacol. 2020;81:105979. 31771816 10.1016/j.intimp.2019.105979

[7] Wu Y , Li Y , Wu B , et al. β‐Arrestin2 inhibits expression of inflammatory cytokines in BEAS‐2B lung epithelial cells treated with cigarette smoke condensate via inhibition of autophagy. Cell Physiol Biochem. 2018;50(4):1270‐1285. 30355935 10.1159/000494586

[8] Kersul AL , Iglesias A , Ríos Á , et al. Molecular mechanisms of inflammation during exacerbations of chronic obstructive pulmonary disease. Arch Bronconeumol. 2011;47(4):176‐183. 21454005 10.1016/j.arbres.2010.12.003

[9] Barnes PJ . Oxidative stress‐based therapeutics in COPD. Redox Biol. 2020;33:101544. 32336666 10.1016/j.redox.2020.101544 PMC7251237

[10] Kirkham PA , Barnes PJ . Oxidative stress in COPD. Chest. 2013;144(1):266‐273. 23880677 10.1378/chest.12-2664

[11] Fischer BM , Voynow JA , Ghio AJ . COPD: balancing oxidants and antioxidants. Int J Chron Obstruct Pulmon Dis. 2015;10:261‐276. 25673984 10.2147/COPD.S42414 PMC4321570

[12] Maremanda KP , Sundar IK , Rahman I . Role of inner mitochondrial protein OPA1 in mitochondrial dysfunction by tobacco smoking and in the pathogenesis of COPD. Redox Biol. 2021;45:102055. 34214709 10.1016/j.redox.2021.102055 PMC8258692

[13] Prakash YS , Pabelick CM , Sieck GC . Mitochondrial dysfunction in airway disease. Chest. 2017;152(3):618‐626. 28336486 10.1016/j.chest.2017.03.020 PMC5812762

[14] Cloonan SM , Glass K , Laucho‐Contreras ME , et al. Mitochondrial iron chelation ameliorates cigarette smoke‐induced bronchitis and emphysema in mice. Nat Med. 2016;22(2):163‐174. 26752519 10.1038/nm.4021 PMC4742374

[15] Mizumura K , Cloonan SM , Nakahira K , et al. Mitophagy‐dependent necroptosis contributes to the pathogenesis of COPD. J Clin Invest. 2014;124(9):3987‐4003. 25083992 10.1172/JCI74985 PMC4151233

[16] Zhang M , Zhang Y , Roth M , et al. Sirtuin 3 inhibits airway epithelial mitochondrial oxidative stress in cigarette smoke‐induced COPD. Oxidative Med Cell Longev. 2020;7582980. 10.1155/2020/7582980 PMC7503124 33005288

[17] Zhang M , Tang J , Li Y , et al. Curcumin attenuates skeletal muscle mitochondrial impairment in COPD rats: PGC‐1α/SIRT3 pathway involved. Chem Biol Interact. 2017;277:168‐175. 28951138 10.1016/j.cbi.2017.09.018

[18] Wan R , Fan J , Song H , Sun W , Yin Y . Oxygen‐glucose deprivation/reperfusion‐induced Sirt3 reduction facilitated neuronal injuries in an apoptosis‐dependent manner during prolonged reperfusion. Neurochem Res. 2022;47(4):1012‐1024. 35091982 10.1007/s11064-021-03502-y

[19] Wang T , Cao Y , Zheng Q , et al. SENP1‐Sirt3 signaling controls mitochondrial protein acetylation and metabolism. Mol Cell. 2019;75(4):823‐834.e5. 31302001 10.1016/j.molcel.2019.06.008

[20] Rehan M , Kurundkar D , Kurundkar AR , et al. Restoration of SIRT3 gene expression by airway delivery resolves age‐associated persistent lung fibrosis in mice. Nat Aging. 2021;1(2):205‐217. 34386777 10.1038/s43587-021-00027-5 PMC8357317

[21] Lombard DB , Alt FW , Cheng HL , et al. Mammalian Sir2 homolog SIRT3 regulates global mitochondrial lysine acetylation. Mol Cell Biol. 2007;27(24):8807‐8814. 17923681 10.1128/MCB.01636-07 PMC2169418

[22] Ansari A , Rahman MS , Saha SK , Saikot FK , Deep A , Kim KH . Function of the SIRT3 mitochondrial deacetylase in cellular physiology, cancer, and neurodegenerative disease. Aging Cell. 2017;16(1):4‐16. 27686535 10.1111/acel.12538 PMC5242307

[23] Wang Q , Li L , Li CY , Pei Z , Zhou M , Li N . SIRT3 protects cells from hypoxia via PGC‐1α‐ and MnSOD‐dependent pathways. Neuroscience. 2015;286:109‐121. 25433241 10.1016/j.neuroscience.2014.11.045

[24] Rauf A , Olatunde A , Imran M , et al. Honokiol: a review of its pharmacological potential and therapeutic insights. Phytomedicine. 2021;92:153769. 34597906 10.1016/j.phymed.2021.153769

[25] Ramesh S , Govindarajulu M , Lynd T , et al. SIRT3 activator Honokiol attenuates β‐amyloid by modulating amyloidogenic pathway. PloS One. 2018;13(1):e0190350. 29324783 10.1371/journal.pone.0190350 PMC5764272

[26] Yang J , Wu W , Wen J , et al. Liposomal honokiol induced lysosomal degradation of Hsp90 client proteins and protective autophagy in both gefitinib‐sensitive and gefitinib‐resistant NSCLC cells. Biomaterials. 2017;141:188‐198. 28689115 10.1016/j.biomaterials.2017.07.002

[27] Singh T , Katiyar SK . Honokiol inhibits non‐small cell lung cancer cell migration by targeting PGE₂‐mediated activation of β‐catenin signaling. PloS One. 2013;8(4):e60749. 23580348 10.1371/journal.pone.0060749 PMC3620279

[28] Lv XQ , Qiao XR , Su L , Chen SZ . Honokiol inhibits EMT‐mediated motility and migration of human non‐small cell lung cancer cells *in vitro* by targeting c‐FLIP. Acta Pharmacol Sin. 2016;37(12):1574‐1586. 27593221 10.1038/aps.2016.81 PMC5290996

[29] Park B , Koo SM , An J , et al. Genome‐wide assessment of gene‐by‐smoking interactions in COPD. Sci Rep. 2018;8(1):9319. 29915320 10.1038/s41598-018-27463-5 PMC6006158

[30] Cipollina C , Bruno A , Fasola S , et al. Cellular and molecular signatures of oxidative stress in bronchial epithelial cell models injured by cigarette smoke extract. Int J Mol Sci. 2022;23(3):1770. 35163691 10.3390/ijms23031770 PMC8836577

[31] Yamada K , Asai K , Nagayasu F , et al. Impaired nuclear factor erythroid 2‐related factor 2 expression increases apoptosis of airway epithelial cells in patients with chronic obstructive pulmonary disease due to cigarette smoking. BMC Pulm Med. 2016;16:27. 26861788 10.1186/s12890-016-0189-1 PMC4748455

[32] Bucchieri F , Marino Gammazza A , Pitruzzella A , et al. Cigarette smoke causes caspase‐independent apoptosis of bronchial epithelial cells from asthmatic donors. PloS One. 2015;10(3):e0120510. 25793769 10.1371/journal.pone.0120510 PMC4368206

[33] Chen CH , Li YR , Lin SH , et al. Tiotropium/Olodaterol treatment reduces cigarette smoke extract‐induced cell death in BEAS‐2B bronchial epithelial cells. BMC Pharmacol Toxicol. 2020;21(1):74. 33129351 10.1186/s40360-020-00451-0 PMC7603690

[34] Dong R , Xie L , Zhao K , Zhang Q , Zhou M , He P . Cigarette smoke‐induced lung inflammation in COPD mediated via LTB4/BLT1/SOCS1 pathway. Int J Chron Obstruct Pulmon Dis. 2015;11:31‐41. 26730186 10.2147/COPD.S96412 PMC4694688

[35] Traves SL , Culpitt SV , Russell RE , Barnes PJ , Donnelly LE . Increased levels of the chemokines GROalpha and MCP‐1 in sputum samples from patients with COPD. Thorax. 2002;57(7):590‐595. 12096201 10.1136/thorax.57.7.590 PMC1746378

[36] Gao W , Li L , Wang Y , et al. Bronchial epithelial cells: the key effector cells in the pathogenesis of chronic obstructive pulmonary disease? Respirology. 2015;20(5):722‐729. 25868842 10.1111/resp.12542

[37] Wang H , Yang T , Wang T , et al. Phloretin attenuates mucus hypersecretion and airway inflammation induced by cigarette smoke. Int Immunopharmacol. 2018;55:112‐119. 29245072 10.1016/j.intimp.2017.12.009

[38] Luan G , Zhu Z , Wu K , Yin S . Theaflavin‐3,3′‐digallate attenuates cigarette smoke extract‐induced pulmonary emphysema in mice by suppressing necroptosis. Exp Ther Med. 2022;23(1):11. 34815763 10.3892/etm.2021.10933 PMC8593858

[39] Costa A , Facchini G , Pinheiro ALTA , et al. Honokiol protects skin cells against inflammation, collagenolysis, apoptosis, and senescence caused by cigarette smoke damage. Int J Dermatol. 2017;56(7):754‐761. 28229451 10.1111/ijd.13569 PMC5464984

[40] Hong T , Min H , Hui Z , Yuejian L , Lixing Y , Liang XZ . Oral administration of honokiol attenuates airway inflammation in asthmatic mouse model. Pak J Pharm Sci. 2018;31(4):1279‐1284. 30033411

[41] Van der Toorn M , Rezayat D , Kauffman HF , et al. Lipid‐soluble components in cigarette smoke induce mitochondrial production of reactive oxygen species in lung epithelial cells. Am J Physiol Lung Cell Mol Physiol. 2009;297(1):L109‐L114. 19411310 10.1152/ajplung.90461.2008 PMC2711811

[42] Rom O , Avezov K , Aizenbud D , Reznick AZ . Cigarette smoking and inflammation revisited. Respir Physiol Neurobiol. 2013;187(1):5‐10. 23376061 10.1016/j.resp.2013.01.013

[43] Mao RW , He SP , Lan JG , Zhu WZ . Honokiol ameliorates cisplatin‐induced acute kidney injury via inhibition of mitochondrial fission. Br J Pharmacol. 2022;179(14):3886‐3904. 35297042 10.1111/bph.15837

[44] Liu JX , Shen SN , Tong Q , Wang YT , Lin LG . Honokiol protects hepatocytes from oxidative injury through mitochondrial deacetylase SIRT3. Eur J Pharmacol. 2018;834:176‐187. 30036533 10.1016/j.ejphar.2018.07.036

[45] Zhang Y , Wen P , Luo J , et al. Sirtuin 3 regulates mitochondrial protein acetylation and metabolism in tubular epithelial cells during renal fibrosis. Cell Death Dis. 2021;12(9):847. 34518519 10.1038/s41419-021-04134-4 PMC8437958

[46] Liu J , Zhang T , Zhu J , et al. Honokiol attenuates lipotoxicity in hepatocytes via activating SIRT3‐AMPK mediated lipophagy. Chin Med. 2021;16(1):115. 34758848 10.1186/s13020-021-00528-w PMC8579168

[47] Dong JX , Zhao GY , Yu QL , et al. Mitochondrial dysfunction induced by honokiol. J Membr Biol. 2013;246(5):375‐381. 23595822 10.1007/s00232-013-9543-x

[48] Sarrica A , Kirika N , Romeo M , Salmona M , Diomede L . Safety and toxicology of Magnolol and Honokiol. Planta Med. 2018;84(16):1151‐1164. 29925102 10.1055/a-0642-1966

[49] Hammad A , Namani A , Elshaer M , Wang XJ , Tang X . "NRF2 addiction" in lung cancer cells and its impact on cancer therapy. Cancer Lett. 2019;467:40‐49. 31574294 10.1016/j.canlet.2019.09.016

[50] Xu K , Ma J , Hall SRR , Peng RW , Yang H , Yao F . Battles against aberrant KEAP1‐NRF2 signaling in lung cancer: intertwined metabolic and immune networks. Theranostics. 2023;13(2):704‐723. 36632216 10.7150/thno.80184 PMC9830441

[51] Cuadrado A , Rojo AI , Wells G , et al. Therapeutic targeting of the NRF2 and KEAP1 partnership in chronic diseases. Nat Rev Drug Discov. 2019;18(4):295‐317. 30610225 10.1038/s41573-018-0008-x

